# The trauma of ongoing conflict and displacement in Chechnya: quantitative assessment of living conditions, and psychosocial and general health status among war displaced in Chechnya and Ingushetia

**DOI:** 10.1186/1752-1505-1-4

**Published:** 2007-03-13

**Authors:** Kaz de Jong, Saskia van der Kam, Nathan Ford, Sally Hargreaves, Richard van Oosten, Debbie Cunningham, Gerry Boots, Elodie Andrault, Rolf Kleber

**Affiliations:** 1 Médecins Sans Frontières, Plantage Middenlaan 14, 1018 DD Amsterdam, The Netherlands; 2 Department of Clinical Psychology, Utrecht University, The Netherlands

## Abstract

**Background:**

Conflict in Chechnya has resulted in over a decade of violence, human rights abuses, criminality and poverty, and a steady flow of displaced seeking refuge throughout the region. At the beginning of 2004 MSF undertook quantitative surveys among the displaced populations in Chechnya and neighbouring Ingushetia.

**Methods:**

Surveys were carried out in Ingushetia (January 2004) and Chechnya (February 2004) through systematic sampling. Various conflict-related factors contributing to ill health were researched to obtain information on displacement history, living conditions, and psychosocial and general health status.

**Results:**

The average length of displacement was five years. Conditions in both locations were poor, and people in both locations indicated food shortages (Chechnya (*C*): 13.3%, Ingushetia (*I*): 11.3%), and there was a high degree of dependency on outside help (*C*: 95.4%, *I*: 94.3%). Most people (*C*: 94%, *I*: 98%) were confronted with violence in the past. Many respondents had witnessed the killing of people (*C*: 22.7%, *I*: 24.1%) and nearly half of people interviewed witnessed arrests (*C*: 53.1%, *I*: 48.4%) and maltreatment (*C*: 56.2%, *I*: 44.5%). Approximately one third of those interviewed had directly experienced war-related violence. A substantial number of people interviewed – one third in Ingushetia (37.5%) and two-thirds in Chechnya (66.8%) – rarely felt safe. The violence was ongoing, with respondents reporting violence in the month before the survey (*C*: 12.5%, *I*: 4.6%). Results of the general health questionnaire (GHQ 28) showed that nearly all internally displaced persons interviewed were suffering from health complaints such as somatic complaints, anxiety/insomnia, depressive feelings or social dysfunction (*C*: 201, 78.5%, CI: 73.0% – 83.4%; *I*: 230, 81.3%, CI: 76.2% – 85.6%). Poor health status was reflected in other survey questions, but health services were difficult to access for around half the population (*C*: 54.3%, *I*: 46.6%).

**Discussion:**

The study demonstrates that the health needs of internally displaced in both locations are similarly high and equally unaddressed. The high levels of past confrontation with violence and ongoing exposure in both locations is likely to contribute to a further deterioration of the health status of internally displaced. As of March 2007, concerns remain about how the return process is being managed by the authorities.

## Background

The conflict in Chechnya has resulted in over a decade of violence, human rights abuses, criminality and poverty. Since the start of the second war between Chechnya and Russia in 1999, thousands of civilians have been killed or have disappeared, all in a climate of impunity.

Years of conflict have resulted in severe destruction of health infrastructure. Many doctors have left the country, while those who remain in Chechnya often fear for their personal safety. Lack of experienced medical personnel, especially in remote rural districts, is one of the biggest problems facing Chechnya's health system today.

The last decade of conflict in Chechnya resulted in around 260,000 Chechens being displaced to neighbouring Ingushetia, most finding shelter in tent camps and collective squats (*Kompakniki*) or spontaneous settlements – farms, sheds, train wagons, and factories. Living conditions in tent camps and spontaneous settlements have been poor. In a 2003 survey carried out by Médecins Sans Frontières (MSF) [1], 54% of the families interviewed in tent camps in Ingushetia stated that their tents leaked, did not have protection from the cold, or had no flooring in conditions where temperatures regularly fall bellow -20°C.

The Ingushetian and Russian governments have increased pressure on the Chechen displaced population to repatriate. Physical, psychological and administrative harassment, the cutting-off of basic services such as gas, water and electricity, and intense propaganda about imminent camp closures, were all used to compel people to return to Chechnya [2]. 'Repatriation' was pushed forward despite the fact that people did not want to return to Chechnya due to the continuation of the conflict and insecurity, and the lack of proper shelter and adequate health services in Chechnya.

To inform the future direction of assistance programmes MSF undertook quantitative surveys among the displaced populations on both sides of the border – both in the spontaneous settlements in Ingushetia and temporary accommodation centres (TACs) housing returned internally displaced within Chechnya. As a consequence of poor health infrastructure and limited external assistance, the health status of internally displaced in Chechnya and Ingushetia is poorly documented; to our knowledge no systematic data on the general and psychosocial health status of this population have been previously published.

## Assessment of violence and related health needs

### Methods

Two surveys were executed: one in Ingushetia (January 2004) and one in Chechnya (February 2004). A systematic sampling method was applied in both locations [3]. Sample size was based on an estimated prevalence of trauma-related psychological problems of 20% [4], a precision of 5% (confidence interval 95%), and an assumed dropout rate (including refusal) of 5%. This gives a samples size of 257 households in each location.

Official demographic data were used to calculate the sampling interval. In Ingushetia, the population to be surveyed was divided over 143 spontaneous settlements (tent-like arrangements within empty buildings). The official population was 21,901 with an average household size of 5.3 persons distributed over 4107 households. In order to arrive at a sample size of 257, a sampling interval of 15.9 was required (rounded to 16).

In Chechnya, the target population was those living in 20 TACs. According to the authorities approximately 3,520 households were permanently present in the TACs. Given the average household size of 5.7, the population was estimated at 20,064. In order to arrive at a sample size of 257, a sampling interval of 13.7 was used (rounded to 14).

In both places the number of interviews per settlement (or TAC) was proportionally related to the number of inhabitants (a logical result of a systematic sampling). The first household was randomly chosen to start the survey in each location. The first household for the systematic sample in the TACs (Chechnya) was chosen randomly by taking a random number from the sampling interval and choosing the house with that number. The next households were chosen according to the fixed sampling interval (14) following a specific direction. Households in the spontaneous settlements in Ingushetia were not systematically ordered, so the starting household was randomly chosen by spinning a pen in the centre of the settlement and the survey started with the first household in that direction. The next household was chosen in a predefined circular direction (systematic) according to the sampling interval (16).

Only people aged 18 or above were interviewed. To avoid selection bias a coin was tossed before knocking on the door to determine whether a male or female respondent would be requested. If the person answering the door was the opposite gender to that determined for selection, the interviewer asked whether there was a respondent of opposite gender and the same age in the household. If no one of the desired gender was present the person answering the door was interviewed. If nobody answered the door the adjacent household was selected.

All interviews were done during the day, with an average of four interviews conducted daily by each team member. Interviews lasted a maximum of 60 minutes and for those participants that needed follow-up support, referral to professional counsellors was offered.

All participants gave written permission for their participation. Interviewers respected confidentiality at all times; guarantees of anonymity were given to each participant, together with a clear explanation of the purpose of the survey and the fact that the general findings would be released publicly. It was made clear to participants that they would not receive any compensation for participating in the survey, and that they could decide at any moment to stop the interview without giving a reason.

Forms were registered anonymously and data were analysed by EXCEL and EPIINFO-6 using descriptive and univariate analyses.

### Instruments

The survey questionnaire was translated from English into both Russian and Chechen, and then back translated to English, and differences discussed and agreed on. The design of the questionnaire was informed by experiences from other assessments done in acute conflict settings [5,6]. Triangulation (the use of different sources and/or methods to verify validity when information is potentially conflicting or inconsistent [7]) of several conflict and health-related variables and methods (open-ended questions, semi-structured questionnaires) were used to get insight in the suffering and needs of the Chechnen IDPs in both Ingushetia and Chechnya.

#### Demographics

General demographic data (age, gender etc.) were obtained.

#### Displacement history

Questions on displacement history were asked in order to seek insight into the collective experience of being displaced and their wishes to leave the settlements and preferred locations of return.

#### Living circumstances

Several questions on the availability of water and sanitation, food and physical shelter were posed.

#### Confrontation with violence

People are confronted with traumatic events in several ways, including exposure to an event (being in the area but not witnessing or self-experiencing an event), witnessing of an event (seeing the event happen) and self-experience. All are established risk factors for developing health (including mental health) problems [8-10]. Generally speaking the proximity to the event [11-13], the severity of the incident [14], and the extent of the physical injury increases the risk of developing health problems. A list of violent events was developed in close consultation with the national counselling staff. Both the composition of the list and the outcomes provide an important testimony of the collective experience of violence.

A distinction was made between recent (i.e. the previous month) and past (since the start of the conflict in 1994) experiences for two reasons. First, it gives insight in the current security situation. Secondly, it gives an indication of the number of potentially traumatic events experienced over time (accumulation) as long-term exposure to violence is a risk factor for developing health problems [15].

#### Loss

In addition to questions relating to violence, questions on the consequences of the conflict such as human and material loss were included.

#### General Health

The General Health Questionnaire 28 [16] (GHQ 28) is a tool that has been widely used for many years to screen general health in community settings including those affected by violence [17]. Four subjective indicators of health are assessed: somatic complaints, anxiety and insomnia, social dysfunction, and depressive feelings. These subscales are not designed to make a specific diagnosis for an individual, and are not mutually independent [18]. However, for assessment of general health of a community it is helpful to identify subscales that are proportionally higher than others. For each of the 28 items, one of four answers is proposed: less than usual; usual; more than usual; and much more than usual (Likert scale).

People suffering from chronic or traumatic stress often report non-specific complaints such as headaches, stomach problems, general body pain, dizziness or palpitations [19,20]. Open questions were used in this survey to find out the type and order of importance of the subjective health complaints over the past 6 months (maximum of four) in order of priority. All answers on these open questions were then grouped in categories based on prevalence. Closed questions were used to gain information about the availability and accessibility of medical services and drugs. Answers to these questions were registered using a Likert scale.

#### Coping mechanisms

Questions were included that were designed to obtain qualitative information regarding how the respondents coped with their problems.

#### General items

The last section of the questionnaire was used to find out whether respondents were able to distinguish between psychiatric disorders and psychological complaints caused by violence. We included open questions in which respondents were asked to indicate a maximum of four signs of each.

At the end of the survey, we asked respondents what additional support they needed.

## Results

In the following reporting of findings, the Chechen Temporary Accommodation Centres (TACs) and the Ingushetian spontaneous settlements (*Kompakniki*) are shown in the text by using: '*C*' for Chechnya and '*I*' for Ingushetia.

### Demographics

256 people in Chechnya and 283 people in Ingushetia were interviewed. None of those approached for interviewing refused and no interviews were interrupted (i.e. 100% completion). The vast majority of interviewees were Chechen; despite randomisation more females were interviewed then men (*C*: 70.3%, 180; *I: 65.4%*, 185). To a lesser extent females were also over-represented in the general population (*C*: 52.5%, *I: 55.4%*).

### Displacement

Displacement mainly occurred in two periods, consistent with periods of severe conflict in Chechnya: 1994/1995 and 1999/2000. The majority of those interviewed had been displaced for at least four years and had changed location between two and five times. Most participants indicated a wish to return to their place of origin. The two groups stated different reasons for not returning. For those living in Chechnya lack of shelter was the main reason for not returning to their hometown (200, 78.4%) while insecurity was less important (25, 9.8%). For those interviewed in Ingushetia insecurity was rated much higher (139, 49.1%) and lack of shelter (129, 45.6%) was rated lower.

The main stated reason for those who left Ingushetia to live in the Chechnen TACs were: the poor living circumstances in the spontaneous settlements, homesickness and the prospect of compensation offered by the authorities.

### Living conditions

In Ingushetia, lack of proper shelter (*C*: 11, 4.3%, *I*: 108, 38.2%) and inability to keep warm (*C*: 47, 18%, *I*: 113, 40%) was reported more frequently than in the Chechnen settlements. The two sites were equally poor in terms of toilet facilities (*C*: 184, 72.4%, *I*: 255, 90.1%) and food was a problem for one in ten (*C*: 34, 13.3%, *I*: 32, 11.3%) Almost all respondents were dependent on charity. It should be noted that while the TACs were intended for short stay only, a substantial number of people had been there for one to two years (87, 34.1%, n = 255), or longer (33, 12.9%).

### Confrontation with violence

#### Month prior to the survey

Nearly twice as many people in Chechnya (C: 171, 66.8%, *I*: 106, 37.5%) indicated that they never or only occasionally felt safe (Table 2). A similar difference was found with respect to exposure to conflict-related violence in the last month: one in ten people (32; 12.5%) in Chechnya said they had been affected, reporting over 60 violence-related events. Most frequently mentioned were: mopping up operations (often violent operations used by the army to identify 'terrorists' among the civilian population) (22 occurrences) and to a lesser extent attacks and crossfire (both more then 8 occurrences). In Ingushetia fewer people (13, 4.6%) reported exposure to violence in the past month (31 violent events). Most of these incidents (25) were reported as being self-experienced by the participants (several participants experienced more than one event). For the majority (18 occurrences) of these cases the person interviewed had been detained/taken hostage.

**Table 2:** experience of traumatic incidents occurring in the month before the survey

	**Chechnya**		**Ingushetia**	
	**n = 256**	**%**	**n = 283**	**%**

Fears for personal safety	171	66.8%	106	37.5%
Exposure to violence	32	12.5%	13	4.6%
Directly targeted by violence themselves	4	1.6%	25	8.8%
Loss of nuclear family member^i ^in past 2 months	19	7.4%	24	8.5%

i) Family = nuclear family (parents and their children)

#### Since start of the conflict

Exposure to violence since the start of the conflict was similar for both groups in Chechnya and Ingushetia (*C*: 241, 94%, *I*: 5, 98%). The most common events (Table 3) included mopping-up operations, aerial bombardment, mortar fire, attack on house or village, crossfire, burning of houses, and destruction of property.

**Table 3:** Overview of participants' experience of traumatic incidents occurring since the start of the conflict (1994). (Participants could report more then one event)

	**Chechnya**		**Ingushetia**	
	**n = 256**	**%**	**n = 283**	**%**

**Exposure to violent events**				
No exposure to conflict	15	6%	5	2%
Aerial bombardments	206	80.5%	220	77.7%
Mopping-up operations	206	80.5%	217	76.7%
Mortar fire	183	71.5%	194	68.6%
Attack on house/village	178	69.5%	205	72.5%
Cross-fire	158	61.7%	170	59.7%
Taking risks to find food	119	46.5%	125	44.2%
Burning of houses	114	44.5%	117	41.3%
**Witnessed events**				
Arrests	136	53.1%	137	48.4%
Maltreatment	144	56.2%	126	44.5%
Killings	58	22.7%	68	24.1%
Torture	14	5.4%	16	5.6%
Rape	2	0.8%	7	2.5%
Known instances of rape	181	71.1%	204	72.1%
**Self-experienced events**				
Maltreatment	66	25.8%	58	20.5%
Detention and hostage	25	9.8%	27	9.5%
Kidnapped	18	7%	21	7.4%
Forced labour	15	5.8%	23	8.1%
Torture	7	2.7%	11	3.9%
Injured by mine	1	0.4%	5	1.8%
**Arrests/disappearances:**				
*Nuclear family*	57	22.3%	54	19.1%
*Extended family, friend, neighbour*	71	27.7%	65	23.0%
*Friend neighbour*	149	58.2%	118	41.7
*Other*	126	49.2%	108	38.2
**Material losses**				
Loss of house	249	97.3%	250	88.3%
Loss of all possessions	254	99.2%	268	94.7%

Respondents from Chechnya and Ingushetia witnessed a similar number of violent events. More than one in five witnessed the killing of people (*C*: 58, 22.7%, *I*: 68, 24.1%) and nearly half had witnessed maltreatment (*C*: 144, 56.2%, *I*: 126, 44.5%). Several people had been witness to torture (*C*: 14, 5.4%, *I*: 16, 5.6%). While many people had heard about incidences of rape (*C*: 181, 71.1%, *I*: 204, 72.1%), only a few had witnessed it (*C*: 2, 0.8%, *I*: 7, 2.5%).

In Chechnya 88 (34.4%) respondents had personally experienced violence since the onset of the conflict. In Ingushetia this was slightly lower, at 80 (28.3%). The type of self-experienced violence was similar in both locations, the most frequently reported events being maltreatment, detention, arrest, and forced labour. Torture and mine injuries were also reported. Disappearances among members of the nuclear family (partners, siblings) affected one fifth of the interviewees (*C*: 57, 22.3%, *I*: 54, 19.1%).

### Loss

#### Material loss

Nearly all respondents reported losing all possessions including their house (*C*: 254, 99.2%, *I*: 268, 94.7%).

#### Mortality in the previous two months

In Chechnya nineteen participants (7.4%) reported 28 deaths in their nuclear family over the past two months (see Table 4). Eleven of them (39.2%, n = 28) were reported as being violence-related such as mine accidents, terrorist acts, and bombardments. In Ingushetia 24 people (8.5%) reported 26 deaths in their nuclear family (Table 4). Five of these deaths were violence-related (19.2%, n = 26). The majority (*C*: 18, 64.3%, n = 28; *I*: 17, 65.4%, n = 26) of deaths were among males.

**Table 4:** Human loss reported by participants

	**Chechnya**		**Ingushetia**	
	**n = 256**	**%**	**n = 283**	**%**

***Mortality in the 2 months preceding the survey***				

Loss of nuclear family memberii in past 2 months	19	7.4%	24	8.5%

***Mortality since the start of the conflict***				

**Reported Deaths (classified by participants relationship to individual affected)**				
Nuclear family (parents, children, siblings)	101	39.5%	95	33.6%
- Witnessed*	35	13.7%	38	13.4%
Extended family	107	41.8%	112	39.6%
- Witnessed*	20	7.8%	29	10.3%
Friend/Neighbour	189	73.8%	200	70.7%
- Witnessed*	27	10.5%	32	11.3%
Other	163	63.7%	155	54.8%
- Witnessed*	20	7.8%	25	8.8%

* Indicates participant directly witnessed the reported death

ii) Family = nuclear family (parents and their children)

#### Mortality since the start of the conflict

Since the start of the conflict one third of the respondents in both Chechnya and Ingushetia (*C*: 101, 39.5%, *I*: 95, 33.6%) reported the loss of at least one nuclear family member (Table 4). Over two-thirds of people had lost a friend and/or neighbour (*C*: 189, 73.8%, *I*: 200, 70.7%). Many respondents actually witnessed the violent death of those close to them.

### General Health

#### General Health Questionnaire

**Figure 1:**
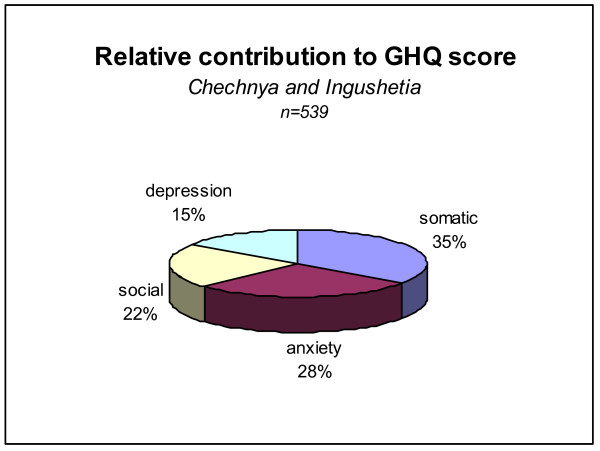
Outcomes of the General Health Questionnaire.

The GHQ 28 was found to be well accepted and easy to administer, but has not been validated for the Caucasus so results must be interpreted with caution (see Discussion). Using the standard cut-off score of 5 [16], it was found that almost everyone could be considered to be at risk of ill health (*C*: 253, 98.8%, CI: 96.6% – 99.8%; *I*: 278, 98.2%, CI: 95.9% – 99.4%). When the cut off score was raised to 11 (the average mean found in a similar study done following the Kosovar conflict [17]) still around 80% of the population was found to be at risk (*C*: 201, 78.5%, CI: 73.0% – 83.4%; *I*: 230, 81.3%, CI: 76.2% – 85.6%). The subscale (Figure 1) on somatic symptoms (*C*: 36%, *I*: 34%) is the largest contributor to high GHQ scores, followed by anxiety (*C*: 27%, *I: 28%*) in both populations.

#### Subjective health reports

The majority of respondents indicated feeling often (*C*: 131, 51.4%, *I*: 171, 60.3%) or sometimes (*C*: 78, 30.6%, *I*: 74, 26.2%) unhealthy in the past six months (Table 5). Respondents indicated to have an average of 2.6 (*C*) and 2.7 (I) symptoms at the time of interview (*C*: 659; *I*: 752, maximum four per participant). A considerable number of respondents indicated cardiovascular problems (*C*: 173, 26.3%, *I*: 89, 11.8%); headaches were the second most frequently reported complaint (*C*: 135, 20.5%, *I*: 160, 21.3%). Muscle or joint pain, chronic disease, nervous complaints and stomach complaints were also reported.

**Table 5:** Self reported health and health complaints over the past six months (maximum four complaints per participant).

	**Chechnya**		**Ingushetia**	
**Subjective (self reported) health**	**n = 255**	**%**	**n = 282**	**%**

Often feeling unhealthy in general	131	51.4%	171	60.3%
Sometimes	78	30.6%	74	26.2%
Rare	36	14.1%	28	9.9%
Health not a concern	10	3.9%	9	3.2%
				

**Health problems experienced in last 6 months (percentages from total number of complaints)**	**n = 659 complaints**	**%**	**n = 752 complaints**	**%**

Cardiovascular	173	26.3%	89	11.8%
Headache	135	20.5%	160	21.3%
Muscle/joint pain	73	11.1%	198	26.3%
Chronic diseases	92	14%	85	11.3%
Nervous complaints	65	9.9%	55	7.3%
Stomach complaints	41	6.2%	55	7.3%
Other	80	12.1%	110	14.6%

#### Availability and accessibility health services and drugs

A considerable number indicated that medical services were rarely (*C*: 96, 37.5%; *I*: 77, 27.2%) or not at all accessible (*C*: 43, 16.8%; *I*: 55, 19.4%). Over half reported difficulties in accessing drugs, stating they were rarely (*C*: 92, 35.9%; *I*: 85, 30.0%) or never available (*C*: 66, 25.8%; *I*: 70, 24.7%).

### Coping mechanisms

Most respondents believed the conflict had triggered mental disturbance or feelings of being upset (*C*: 205, 80.1%; *I*: 189, 66.8%). To cope with their psychological distress people responded that their first most important coping strategy was 'turning their head' (a local term meaning to deny a problem exists) (*C*: 123, 48.1%, *I*: 131, 46.3%). In the second response category the preferred option was prayer (*C*: 137, 53.5%, *I*: 131, 46.3%). A third and last stated option was the support of the family members (Table 6).

**Table 6:** Coping mechanisms of the participants (maximum of three answers possible)

**Managing stress**	**Chechnya**		**Ingushetia**	
***First mentioned***	**n = 256**	**%**	**n = 283**	**%**

'Turn my head' (see footnote vii)	123	48.1%	131	46.3%
-Keep busy	50	19.5%	59	20.9%
-Aggressive behaviour	56	21.9%	51	18%
-Praying	27	10.5%	40	14.1%
-Other			2	0.7

***Second mentioned***	**n = 256**	**%**	**n = 283**	**%**

Praying	137	53.5%	131	46.3%
-Aggression	40	15.6%	46	16.3%
-Talking	32	12.5%	40	14.1%
-Keep busy	24	9.4%	46	16.3%
-Drug/alcohol use	13	5.1%	26	9.2%
- Other	10	3.9%	10	3.5%

***Third mentioned***	**n = 255**	**%**	**n = 220**	**%**

Support of family members	101	39.6%	106	48.2%
-Talking to others	66	25.9%	7	3.2%
-Drug/alcohol use	17	6.7%	28	12.7%
-Other	71	27.8	43	19.6%

Suicide is considered a sin in the Muslim religion (as in many other societies) and therefore a taboo subject. Nevertheless, nearly one in ten respondents (*C*: 21, 8.2%; *I*: 28, 9.9%) knew somebody who had attempted suicide (although several respondents could be referring to the same incident).

### General items

When asked what advice respondents could give MSF regarding its activities most responses advised MSF increasing their counselling activities (*C*: 81, 31.6%; *I*: 114, 40%). Some suggested MSF increase its medical activities (*C*: 50, 19.5%; *I*: 27, 9.5%). Notably, a number of people wanted MSF to advocate on their behalf (*C*: 38, 14.8%; *I*: 53, 18.7%).

## Discussion

To our knowledge this is the first publication of the general and psychosocial health status of Chechnen's internally displaced. The self-reported health conditions and the general health questionnaire showed high levels of medical and psychosocial needs. Access to health care (including mental health) was poor in both locations. The most frequently used coping mechanisms for psychological distress (denying the problem, praying, support of family members) did not seem to be effective. Living conditions in the Ingushetian spontaneous settlements were rated worse while people in the Chechnen TACs had more security problems (feeling less safe, more incidents in the last month, most violent deaths in the last two months).

Our findings on the General Health Questionnaire 28 (GHQ 28) [18] indicated that nearly all IDPs were suffering from health complaints such as somatic complaints, anxiety/insomnia, depressive feelings or social dysfunction when applying the recommended cut-off score for this questionnaire. Even when a higher cut-off score was set, still around 80% of respondents were found to suffer from general health problems. This is substantially higher than findings from elsewhere: for example a study from Iran using the same instrument (with a normal cut off) found a prevalence of 17% [21]. Subjective health impressions further confirmed the poor general health found in the GHQ 28, with half of respondents in both locations reporting to often feel unhealthy. Also, the average number of complaints pointed in the same direction.

The types of complaints reported are associated with a high level of (traumatic) stress, with non-specific physical signs like headaches and muscle/joint/body pain commonly reported [18]. Cardiovascular complaints represent one quarter of all complaints mentioned; however, to what degree these are linked to the stress or the general situation of conflict is unclear, as incidence of cardiovascular complaints in the former Soviet Union is generally high.

For displaced populations, the length of stay in temporary (and often precarious) accomodation is associated in other studies with higher likelihood of developing symptoms of psychological distress [22-24]. The average length of being displaced in both locations was five years. Most people had to move at least two times.

Chronic exposure to traumatic events is associated with higher levels of mental health problems and poorer physical health [25,26], and witnessing and self-experienced extreme violence is also associated with psychosocial and mental health problems, including depression [27], generalised anxiety disorder [30], and post-traumatic stress disorder [11,12,31,32]. Both survey groups had experienced similar levels of violence since the start of the conflict (exposure, witnessed, self-experienced), possibly contributing to ill health outcomes.

Nearly all of the people interviewed wished to return to their place of origin. In Chechnya, lack of shelter was the main reason for not returning; in Ingushetia, insecurity was the most important concern. This difference may be explained by the fact that for people in Chechnya insecurity was a daily reality which cannot be changed, whereas for those in Ingushetia the security situation in Chechnya was perceived as a threat to avoid.

Caution is required to avoid facile labelling the survey population with physical or mental diagnoses. There is a tendency to report on the mental health consequences in terms of psychiatric or psychological disorders often using post-traumatic stress disorder (PTSD) as the pathway to show the mental health consequences of war. It is incorrect to reduce the experience of conflict and violence to the individual using bio-psycho-medical terminology [33], and it may be unnecessarily stigmatising to label someone with PTSD when PTSD which is not the only possible disorder that can result from a traumatic event, even according to the DSM IV system (Diagnostic Statistic Manual for Mental Health  Disorders number IV, [34]). Co-morbidity, most notably depression [29] and generalised anxiety disorder [30,35,36] has been found to be more prominent in trauma-affected people than was originally assumed. Another consideration is that although nearly all people confronted with war will suffer various negative responses such as nightmares, fears, startle reactions and despair, they will not all develop mental disorders. There are individual ways of adapting to extreme stress [4] that should not be overlooked. Lastly, transfer of Western conceptual frameworks of psychological stress and mental disorders to different countries and cultures is problematic [37].

Nevertheless, attention must be paid to stress and distress in the survey population since prolonged states of either can cause changes in patterns of living that are associated with physical and mental damage [19,38]. The need for health (including mental health) support is further indicated by the fact that over a third of respondants in both locations indicated that MSF should increase their counselling activities. In response to these findings, MSF began a psychosocial intervention in the TACs in Chechnya in February 2004.

### Possible limitations to the survey

The sampling method has been satisfactory. Despite the sensitivity of the questions the completion rate was high (100%). There are, however, a number of potential limitations that merit consideration.

Compared to the overall population data of the authorities the number of people interviewed in Ingushetia was higher then the planned sample size (283 versus 257) suggesting that population figures given by the government are an underestimation. In both studies women were over-represented despite the sampling procedures. The most plausible reason for this is the timing of the interviews: survey teams only worked during the day, when most males were away from the household trying to find work; however, due to security concerns the survey times were limited to daylight hours. The high number of women may have resulted in an overestimation of health needs as women generally report more frequent health concerns compared to men. However, because of the female bias the values on the GHQ might be somewhat lower for the entire population, the main conclusions remain valid. Another possible consideration is that the survey timing may also have caused selection bias of ill people because they tend to stay home.

The survey has no precedence and therefore the GHQ 28 had not been validated for use in the region. We do not believe that this invalidates its value. Health data were assessed through three different methods (semi-structured, questionnaire, open-ended questions), with all findings pointing in the same direction, and triangulation of information generated from different health related topics (displacement, living conditions, confrontation with violence, loss, general health, coping) together establish a picture of violence-related suffering of those enduring the ongoing conflict on the Caucasus. The use of other approaches such as structured clinical interview and clinical examination would certainly have added weight to the validity of our findings, but for operational security reasons this was not possible.

This survey included historical questions over a long timeframe (1994–2004), in addition to questions in the more recent past (30 or 60 days). Recall bias is always a potential confounding variable, particularly when reporting traumatic events. However, an important recent study [40] has shown that refugees remain consistent in reporting major traumatic events such as those we recorded, with more variability occurring in recall of minor historical details. Thus we believe that this bias does not pose a serious threat to the validity of this study.

The category of questions relating to exposure to violence may have included some events that should have been classed as self-experienced or witnessed events despite instructions to interviewers to exclude them from the exposure category, and this may have caused some over-reporting in the data on exposure to violence. Nevertheless, presentation of all categories including exposure remains relevant because proximity to violence is associated with increased risk of health problems or even pathology [11,31]. The high level of war-related violence is also reflected in hospital admission data. According to hospital statistics around one in 20 admissions (783 out of 15,602) to the hospital and outpatient trauma point in Grozny in 2004 were for war trauma. Of those, 384 (50%) were gunshot wounds and 276 (35%) were mine or other explosive wounds. Around a third of these patients died inhospital.

The findings on rape may be underreported. Sexual violence is a taboo topic in Chechnya, but is known to occur. Other organizations working with Chechen refugees have reported a high incidence of repeated sexual violence. In those surveys, it may be that women only felt free to bring up their experience because they were abroad, far away from potential community repercussions [39]. In our survey many people had heard about incidents of rape but only a few had witnessed it, and only one person reported being raped. According to Muslim and local traditional laws, a raped woman is often stigmatised and her whole family becomes a victim of the rape. A Chechen man will be very unlikely to admit to having been raped [39].

While time was taken to carefully explain the terms used in the questionnaire to both survey staff and respondents, we cannot entirely exclude subjective interpretation by interviewer or interviewee. Specifically, for sexual violence we used in our survey the World Health Organization's definition of sexual violence as being "any sexual act, attempt to obtain a sexual act, unwanted sexual comments or advances, or acts to traffic a person's sexuality, using coercion, threats of harm or physical force, by any person regardless of relationship to the victim, in any setting, including but not limited to home or work" [41]. Given the strict religious and cultural norms on sexuality and the comments of our staff we were confident it was in agreement with the popular understanding among Chechens. Although we have no indications this assumption was wrong it is possible that some respondents used their own interpretation. Nevertheless, more objective definitions of questions relating to sexual violence would be useful for such studies.

Despite these potential limitations the survey provides valuable data on the confrontation with violence-related health problems from a conflict where data are near absent due to non-functioning surveillance systems and limited access for external actors.

### Implications of our findings

Recent developments in the Caucasus have overtaken the situation surveyed in early 2004, with the authorities rapidly closing the spontaneous settlements in Ingushetia and sending the IDPs back to the Temporary Accommodation Centres (TACs) in Chechnya.

Our survey data showed that many who returned to Chechnya from Ingushetia were simply changing their status from being IDPs outside to being IDPs inside Chechnya. The fate of those IDPs accommodated in TACs remains an important longer-term question. As of March 2007 concerns remain about how the authorities manage the return process and whether considerations on the wellbeing and health of this group are being taken into account while planning this process.

International humanitarian assistance is an important external support to the population, both in Ingushetia and in Chechnya. However, the extremely high levels of insecurity threaten the aid operations in the Northern Caucasus: since 1995 more than 50 international humanitarian and  workers have been abducted, and some of them have been murdered. As a result the number of international and national staff working in the region has  been dramatically reduced. Due to the highly insecure context MSF has had to conduct "remote control" (minimal contact) operations in Chechnya with minimal direct expatriate supervision.

More importantly, the Russian authorities must guarantee a safe environment; ensure the protection of civilians, as well as appropriate living conditions (including access to health services, sufficient food, shelter and sanitation) for this displaced population. The international community should pay greater attention to the situation of these vulnerable groups that have been largely ignored for the last decade.

## Competing interests

The author(s) declare that they have no competing interests.

**Table 1:** Overview of demographic and socio-economic findings

	**Chechnya**		**Ingushetia**	
	**n**		**n**	

**Population**				
Total population number in TACs, Spontaneous Settlements (official figures)	20,064		21,901	
Interviewees (one per household)	256		283	
Female interviewees	180	70.3%	185	65.4%
Total number of family members in surveyed households	1107		1668	
Average number of family members in surveyed households (official average in population in brackets)	4.3 (5.7)		5.8 (5.3)	
	**n**	**%**	**n**	**%**
**Displacement history**				
Displaced > 4 years	249	98.0%	272	96.1%
*-During first Chechnen War (1994–1995)*	122	48.0%	104	36.8%
*-During second Chechnen War (1999/2000)*	118	46.5%	154	54.4%
*-Others*	9	3.5%	25	8.8%
Displaced < 4 (missing data)	5 (2)	2.0%	11	3.9%
Displaced more than once (2–5 times)	212	83.1%	160	56.6%
Wish to return home	220	86.3%	243	85.9%
**Origin**				
Chechnya	250	97.7%	253	89.4%
Other	6	2.3%	30	10.6%
**Reason for not returning to place of origin**				
Lack of shelter	200	78.4%	129	45.6%
Insecurity	25	9.8%	139	49.1%
Other	31	12.%	15	5.3%
**Reasons for returning to Chechnen TACs**				
Living circumstances in spontaneous settlements Ingushetia	66	27.5%	-	-
Homesick	60	25.0%	-	-
Compensation offered by authorities	40	16.7%	-	-
Directly forced to return	4	1.7%	-	-
Indirectly forced to return (camps closed in Ingushetia)	28	11.7%	-	-
Hope the situation improves	22	9.2%	-	-
Other (missing data)	10 (26)	3.9% (10.2%)	-	-
**Living Circumstances in the Chechnen TACs, Ingushetian spontaneous settlements**				
Poor shelter against weather	11	4.3%	108	38.2 %
Unable to keep warm	47	18%	113	40%
Poor toilet facilities	184	72.4%	255	90.1%
Insufficient food (defined as on at least 5 days a week, having 1 meal or less per day)	34	13.3%	32	11.3%
Dependence on outside assistance	244	95.4%	267	94.3%
